# Acute disseminated encephalomyelitis (ADEM)-like illness in a pediatric patient following COVID-19 vaccination

**DOI:** 10.1259/bjrcr.20220097

**Published:** 2023-02-20

**Authors:** Kenneth Brock, Susana Creagh Reyes, Christopher Conner, Natalie Gillson, Michael Weiss, Osama Elfituri, Amir Paydar

**Affiliations:** 1 Department of Radiology, Newark Beth Israel Medical Center, Newark, New Jersey, United States; 2 Department of Radiology, HCA Florida Aventura Hospital, Aventura, Florida, United States; 3 Florida Radiology Consultants, Fort Myers, Florida, United States; 4 Department of Pediatric Neurology, Golisano Children’s Hospital of Southwest Florida, Fort Myers, Florida, United States; 5 HealthPark Hospital, Fort Myers, Florida, United States; 6 MedStar Medical Group Radiology, MedStar Washington Hospital, Washington, DC, United States

## Abstract

Since the inception of the COVID-19 pandemic, over 60 cases of acute disseminated encephalomyelitis (ADEM) or ADEM-like clinically isolated syndromes have been linked to COVID-19 infection. However, cases linked to COVID-19 vaccination remain exceptionally rare. To the author’s knowledge, eight published cases of ADEM or ADEM-like clinically isolated syndrome have been described after patients received COVID-19 vaccinations, all of which occurred in adults.

This report details the first documented case of an ADEM-like illness in a pediatric patient, which developed shortly after receiving the Pfizer (Pfizer-BioNTech, Germany) COVID-19 vaccination. The patient made a near complete clinical recovery over 10 days after receiving a 5-day course of intravenous immunoglobulin therapy.

## Introduction

ADEM is a rare autoimmune demyelinating central nervous system disease, typically associated with pediatric patients younger than 15 years of age.^1–3^ However, it can occur at any age. It is classically preceded by recent viral illness or vaccination,^1–3^ with some reports indicating that less than 5% of cases occur following vaccination.^2^ ADEM usually manifests as an acute, monophasic illness with characteristic imaging features that include multifocal subcortical and deep white matter lesions in a bilateral but asymmetric pattern.^1–5^ Lesions may involve both brain and spinal cord, with a typical onset of days to weeks following an infection or vaccination.^2^ The main clinical features involve fever, sensorimotor abnormalities, and encephalopathy.

It has recently been suggested that ADEM cases linked to COVID-19 infection may be associated with older age of onset, and higher rates of morbidity and mortality.^6^ Notwithstanding, poor clinical outcomes have still been documented in the limited number of pediatric ADEM cases associated with COVID-19 infection.^7^ Given the limited number of cases linked to COVID-19 vaccination, it is unknown if such association exists between COVID-19 vaccination, and clinical outcome of ADEM. Given the importance of vaccination in the global response to COVID-19, and uncertainty regarding an ongoing need for periodic booster doses, it is particularly essential to monitor adverse clinical events related to COVID-19 vaccination, including in the less well-studied pediatric population.^8^

## Case report

A 10-year-old female with no past medical history presented to Golisano Childrens Hospital, Fort Myers, Florida, United States on December 21, 2021 with 7 days of progressive lower extremity weakness, paresthesia, and urinary retention. No recent symptoms of infection were reported. Neurological examination showed mild lower extremity hyperreflexia, right lower extremity weakness with inability to ambulate, a mild pronator drift, and a right visual field defect. The patient received her second dose of mRNA-based COVID-19 vaccine 16 days prior to the onset of symptoms.

**Figure 1.**
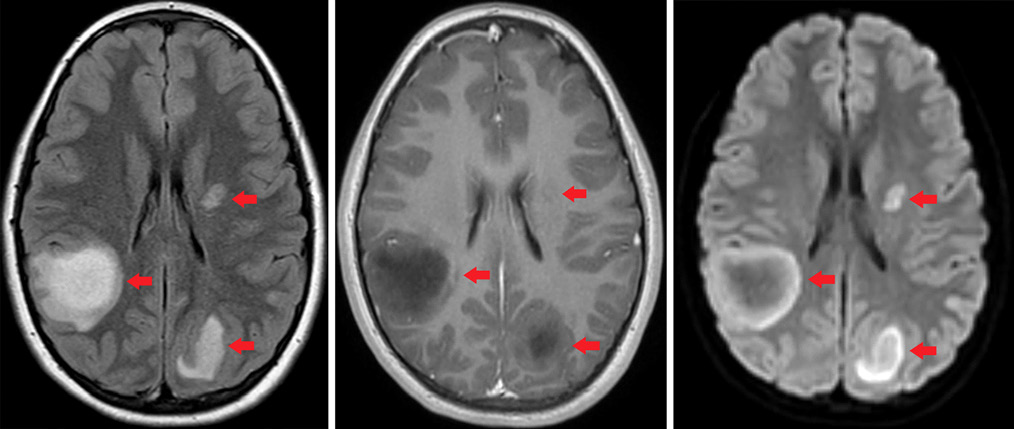
Initial brain MRI findings. Left: Axial FLAIR image demonstrating multifocal supratentorial subcortical and deep white matter lesions (red arrows). Middle: Axial T1 post-contrast image demonstrating peripheral lesion enhancement. Right: Axial DWI image demonstrating avid peripheral diffusion restriction. DWI, diffusion-weighted imaging; FLAIR, fluid attenuation inversion recovery.

**Figure 2.**
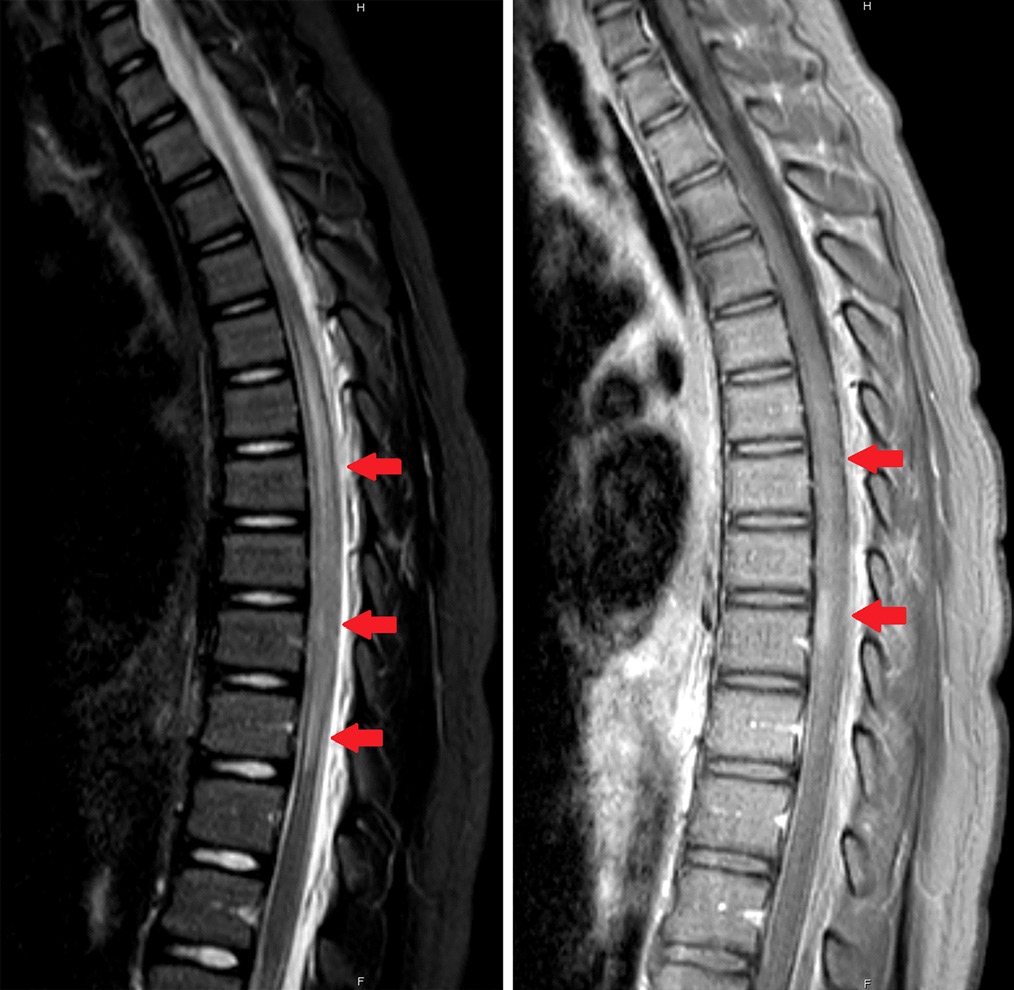
Initial thoracic spine MRI findings. Left: Sagittal STIR image demonstrating multiple longitudinally extensive spinal cord lesions (red arrows). Right: Sagittal T1 post-contrast image demonstrating corresponding lesion enhancement. STIR, short tau inversion recovery.

Upon admission, a comprehensive laboratory assay including CBC, CMP, ESR, and CRP, was negative. A respiratory viral panel which included SARS-COV2 PCR testing was negative. Contrast-enhanced MRI of the brain demonstrated multiple prominent T2/FLAIR hyperintense subcortical and deep white matter lesions with incomplete rim-enhancement, compatible with active demyelination, and avid peripheral diffusion restriction (Figure 1). Contrast-enhanced MRI of the cervical, thoracic, and lumbar spine demonstrated a long-segment, non-expansile, partially enhancing intramedullary lesion within the thoracic spinal cord, also most likely compatible with active demyelinating process (Figure 2). There were no findings suggestive of Guillain-Barré syndrome. Secondary differential considerations included transverse myelitis, CNS lymphoma, and atypical multiple sclerosis.

Lumbar puncture was performed, and CSF analysis showed an isolated leukocytosis with normal protein and glucose levels. A comprehensive autoimmune and infectious CSF panel was obtained, with results pending. On hospital day 2, the patient underwent stereotactic right parietal lobe biopsy, given the diagnostic risk of initiating steroid treatment before fully excluding lymphoma. She then began a 5-day course of i.v. corticosteroids. On hospital day 5, examination showed subtle improvement in motor strength, and resolution of pronator drift. The following day her motor strength continued to improve, and she regained urinary continence. On hospital day 7, CSF autoimmune screening for antibodies associated with demyelinating disorders (including MOG, AQP4, and gangliosides) resulted negative. Microbiology studies (including HSV, VZV, CMV, EBV, and HIV) were also negative, and a CSF flow cytometry showed no evidence of lymphoproliferative neoplasm. On hospital day 8, the patient began intravenous immunoglobulin (IVIG) treatment and transitioned to oral corticosteroids. On hospital day 9, oligoclonal band testing returned normal. By hospital day 10, the patient remained afebrile, hemodynamically stable, and made full recovery of bowel, bladder, and sensory function. Motor strength significantly recovered with physical therapy, but there was a mild, persistent gait unsteadiness. She was safely discharged the same day.

**Figure 3.**
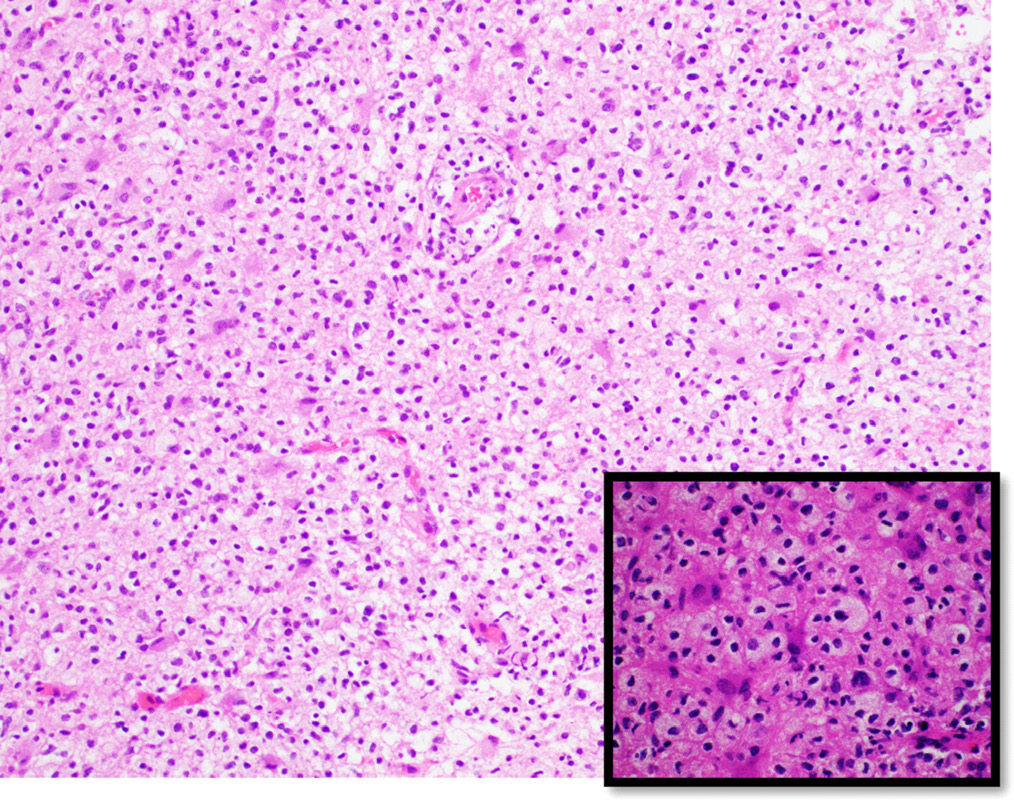
Right occipital lobe brain biopsy demonstrating gliotic cerebral cortex with extensive macrophage-rich inflammatory infiltrate and small lymphocytic component forming perivascular aggregates (hematoxylin and eosin stains, magnification 20x and 60x).

**Figure 4.**
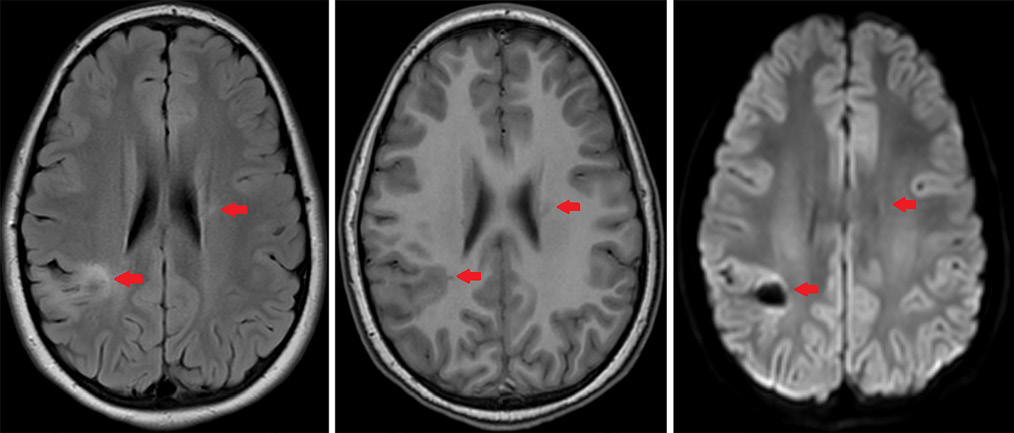
Follow up brain MRI findings. Left: Axial FLAIR image demonstrating decreased FLAIR signal hyperintensity of the white matter lesions (red arrows). Middle: Axial non-contrast T1 image demonstrating lesion signal characteristics relatively isointense to grey matter. Right: Axial DWI image demonstrating decreased diffusion restriction of the lesions. DWI, diffusion-weighted imaging; FLAIR, fluid attenuation inversion recovery.

Three months after initial evaluation, the patient returned for outpatient neurologic follow-up. She reported mild fatigue. Examination showed a mild, persistent gait instability and hip muscle weakness. All other symptoms were resolved. A few days later, final histopathology showed white matter neurons with an extensive macrophage-rich inflammatory infiltrate with small lymphocytic component forming perivascular aggregates (Figure 3). Luxol/H&E stain showed severe loss of myelin, with macrophages demonstrating phagocytosed myelin debris. Immunohistochemistry showed depletion, but relative preservation of axons, with scattered perivascular accumulations of CD3+ T-cells and CD4/CD8 co-expressing T-cells. One month later, a follow-up non-contrast MRI of the brain was performed, since the patient’s parent declined contrast-enhanced MRI. This showed residual, but substantially decreased size and FLAIR signal abnormality of the lesions (Figure 4).

## Discussion

ADEM is a rapidly progressive autoimmune condition that causes multiple demyelinating inflammatory lesions in the brain and spinal cord.^1–5^ The diagnosis should be suspected in a child who develops acute polyneuropathy in the setting of recent infection or immunization.^2^

In our case, these clinical findings improved significantly with corticosteroid and IVIG therapy and are most compatible with ADEM or an ADEM-like clinically isolated syndrome. Notably, the patient did not exhibit encephalopathy, which some authors suggest is a major diagnostic criterion for ADEM.^9^ Whether ADEM or a related clinically isolated syndrome, this disease spectrum is strongly supported by the histopathological findings of patchy inflammatory infiltrates with perivascular lymphocytic aggregates, which Ghali and Young et al observed is more strongly associated with ADEM than other demyelinating diseases.^10,11^

MRI is the imaging modality of choice to evaluate ADEM and helps to distinguish the illness from other similar demyelinating diseases in children such as multiple sclerosis (MS).^2,5^ In ADEM, MRI typically shows multiple bilateral asymmetric T2/FLAIR hyperintense lesions in the subcortical and deep white matter, with relative sparing of the periventricular white matter. The lesions tend to be larger and more numerous than those seen with MS.^2–5^ The thalami and basal ganglia may also be involved. Diffusion restriction is an uncommon imaging finding that suggests a worse prognosis.^3^

In contrast, MS classically shows multiple T2/FLAIR hyperintense lesions that involve the periventricular white matter along the callosal–septal interface, in a more symmetric distribution.^4,5,9^ The lesions in MS typically have better defined margins, while margins are less distinct in ADEM.^5^ Lesions in MS furthermore have a relapsing and remitting course with variable age, while lesions in ADEM are monophasic, typically of the same age, and regress significantly after a short course of corticosteroids.^1–3^ In the spinal cord, MRI shows large confluent intramedullary lesions involving multiple segments in ADEM, while the lesions in MS are smaller and favor the cervical spine^4,5,9^.

A notable exception is the Marburg variant of multiple sclerosis, which often affects younger patients and can be difficult to distinguish from the appearance of ADEM due to fulminant, rapidly progressive demyelination.^4^ The variant can similarly be preceded by fevers and a monophasic course. There are also overlapping MRI features including larger, confluent lesions with tumefactive demyelination and incomplete ring enhancement.^4^ However, unlike ADEM, the peripheral nervous system can be involved.

Notwithstanding, the MRI characteristics of ADEM are typically non-specific, and consequently can be indistinguishable from the appearance of neoplasms such as CNS lymphoma and glioblastoma multiforme.^4,5,12^ Therefore, tissue sampling is usually critical for definitive diagnosis and before safely beginning the appropriate treatment, as was such in our case.

There are little data characterizing a link between COVID-19 vaccination and ADEM. In our case, a similar time course between COVID-19 vaccination and disease onset is observed when compared to other adult cases.^13–20^ Our findings are also congruent with the hypothesis of Rinaldi et al, that T-cell lymphocytic infiltrate found in ADEM pathophysiology is consistent with the ability of COVID-19 vaccines to catalyze a T-cell mediated immune response.^20,21^ Nonetheless, present understanding of both mechanisms remains incomplete.

A total of eight case reports have linked COVID-19 vaccination with ADEM or ADEM-like illness in adults aged 19–84 years old.^13–20^ A ninth adult case was reported in a Korean news article, but limited clinical information is available.^22^ To our knowledge, we report the first known pediatric case of ADEM-like illness associated with COVID-19 vaccination. What substantiates this case is that other infectious and neoplastic etiologies were excluded by imaging and histopathological findings. Several pediatric cases of ADEM linked to COVID-19 infection resulted in poor neurologic outcomes, but it is unclear if such a relationship also exists for COVID-19 vaccination.^7,23^

This case study aims to bring awareness to clinicians of a rare but potentially life-threatening complication of COVID-19 immunization in the pediatric population. It also highlights the role of MRI in supporting the diagnosis of ADEM, differentiating it from similar demyelinating conditions, and assessing treatment response. More data would be needed to identify any statistical correlation between COVID-19 vaccination and pediatric demyelinating disease.

## Learning points

1. Little literature exists suggesting a relationship between COVID-19 vaccinations and acute demyelinating diseases in the pediatric population.
2. Literature suggesting a relationship between COVID-19 vaccinations and acute demyelinating diseases has only pertained to adult patients.
3. This is the first biopsy proven case that suggest a relationship between COVID-19 vaccinations and acute demyelinating diseases in children.
4. This case highlights the pivotal role of MRI in supporting the diagnosis of ADEM and distinguishing it from similar demyelinating conditions such as MS.
